# First Impressions of HIV Risk: It Takes Only Milliseconds to Scan a Stranger

**DOI:** 10.1371/journal.pone.0030460

**Published:** 2012-01-24

**Authors:** Britta Renner, Ralf Schmälzle, Harald T. Schupp

**Affiliations:** 1 Department of Psychology, Psychological Assessment and Health Psychology, University of Konstanz, Konstanz, Germany; 2 Department of Psychology, General Psychology, University of Konstanz, Konstanz, Germany; University of Regensburg, Germany

## Abstract

Research indicates that many people do not use condoms consistently but instead rely on intuition to identify sexual partners high at risk for HIV infection. The present studies examined neural correlates for first impressions of HIV risk and determined the association of perceived HIV risk with other trait characteristics. Participants were presented with 120 self-portraits retrieved from a popular online photo-sharing community (www.flickr.com). Factor analysis of various explicit ratings of trait characteristics yielded two orthogonal factors: (1) a ‘valence-approach’ factor encompassing perceived attractiveness, healthiness, valence, and approach tendencies, and (2) a ‘safeness’ factor, entailing judgments of HIV risk, trustworthiness, and responsibility. These findings suggest that HIV risk ratings systematically relate to cardinal features of a high-risk HIV stereotype. Furthermore, event-related brain potential recordings revealed neural correlates of first impressions about HIV risk. Target persons perceived as risky elicited a differential brain response in a time window from 220–340 ms and an increased late positive potential in a time window from 350–700 ms compared to those perceived as safe. These data suggest that impressions about HIV risk can be formed in a split second and despite a lack of information about the actual risk profile. Findings of neural correlates of risk impressions and their relationship to key features of the HIV risk stereotype are discussed in the context of the ‘risk as feelings’ theory.

## Introduction

As of 2011, around 35 million people are living with HIV, and the worldwide epidemic continues to spread. Between 2001 and 2009, the number of HIV positive people in North America and Western and Central Europe grew by 30% from an estimated 1.8 million to 2.3 million [1]. These figures are particularly troubling because people are now well informed about HIV, its transmission, and effective risk protection measures (i.e. consistent condom use) [1]. However, despite the high level of HIV-related knowledge among the public, various studies observed only inconsistent or even infrequent condom use [2], [3]. In brief, these studies suggest that when it comes to HIV or other sexually transmitted diseases (STDs), knowing the facts is insufficient to motivate consistent protective behavior.

One factor that could explain the gap between common awareness about HIV transmission and the fairly low use of condoms is that condoms are often associated with negative attitudes and feelings (e.g., discomfort or embarrassment) [4]–[9]. This may encourage people to seek ways to circumvent condom use by relying on alternative risk protection strategies. Indeed, field research has shown that a common strategy for circumventing condoms is to screen potential partners for their risk status in order to detect and avoid risky partners [10]. Unfortunately, screening individuals for their risk status is an illusionary risk protection strategy: it is ineffective, fallible, and may reassure people with a false sense of control and encourage a sense of personal invulnerability [8], [10]. Considered from a rational actor point of view, relying on this strategy seems irrational, yet, the evidence shows that this behavior is quite common in real-life circumstances [8]–[13].

Empirical evidence from the field of health psychology and social cognition suggests that the perceived riskiness of potential partners is based on indeterminate person impressions. Interviews and focus group studies showed that people are often convinced that they ‘just know’ whether a person is risky or safe - even when they do not know much about the respective person [11], [12]. Similarly, people who have had unsafe intercourse believed that their partners were safe [11]. Finally, laboratory research has shown that people are overconfident regarding their ability to identify HIV positive individuals and that feelings of risk are based on superficial person characteristics that are unrelated to HIV status [7], [10], [13]–[15]. These findings lead to the question of which trait characteristics inform such intuitive impressions of the ‘riskiness’ or ‘safeness’ of others.

### Intuitive Risk Impressions of Others: Underlying Person Characteristics

Because an STD or HIV infection does not lead to immediate health problems, there are no overt or observable signs that accurately indicate HIV or STD risk status. Thus, impressions about risk status are likely to be inferred from other personal characteristics [10]. From an evolutionary perspective, one could argue that HIV risk might be gauged according to a general ‘positive - negative’ valence evaluation [16]. Specifically, determining whether a person is dangerous or safe represents one of the most fundamental decisions for securing survival and well-being. Research on face perception has shown that faces are spontaneously evaluated according to their perceived attractiveness and trustworthiness [17]. This line of thinking may be extended to the health risk context. Previous research in health psychology has investigated spontaneous avoidance reactions to symptoms of infectious diseases such as pustules and rashes [18]. Similarly, one might propose that perceived healthiness serves as a core attribute for inferences about another's risk status. Moreover, the intuitive perception of another person's HIV risk status might be related to the typical characteristics of people with a high risk of HIV (‘high at risk stereotype’) [19]–[22]; previous has research revealed that a low sense of responsibility is a key characteristic attributed to the high at risk stereotype. Interestingly, this attribute turned out to be as important as the actual risk behavior [22]. To summarize, impressions about the safety or riskiness of others may be related to inferences about general trait characteristics, such as a perceived valence, healthiness, attractiveness, trustworthiness, or responsibility. These dimensions might covary and overlap, and may inform intuitive HIV risk perceptions based on as little evidence as a glance at an unacquainted person. The relationship between these individual trait characteristics and inferences about HIV risk, however, has not yet been investigated.

### Intuitive Risk Impressions of Others: Neural Correlates

In the present research we assumed that impressions of HIV risk arise from intuitive processing [16], [23], [24]. It has been proposed that the intuitive sensing of risk builds on affective processes that may result in subtle experiential changes (i.e. feelings) [16], [24]. In the past decade, research in affective neuroscience has delineated neural correlates of affective processing. Event-related brain potential (ERP) recordings enable researchers to determine the time-course of stimulus processing and affect-induced attentional modulations. The late positive potential (LPP) has been consistently and reliably observed as a cortical marker of affect processing across a wide range of stimulus materials (i.e., natural emotional scenes, facial expressions, and symbolic gestures) [25]–[27]. Building on these findings, a recent study investigated first impressions of unacquainted target persons to determine the neural base of intuitive HIV risk perception [28]. Faces of persons evaluated as being high at risk for HIV infection elicited significantly larger LPPs compared to faces of persons believed to be low at risk. Accordingly, the perception of risky as compared to safe persons elicited the brain signature of affective stimulus evaluation. Furthermore, compared to ‘analytic processing’ [16], [24], intuitive processing is considered to be effortless, non-deliberate, and quick [23], [29]. In line with this contention, the differentiation of risky and safe persons occurred in a split second, preceding the opportunity for systematic reasoning about health risks. These results provide first evidence for the hypothesis of the intuitive perception of HIV risk.

### The Present Study

In order to examine the intuitive basis of HIV risk perceptions, we combined research on person perception with methods from affective neuroscience: First, we sought to determine which trait characteristics (e.g., perceived healthiness, trustworthiness) are associated with perceived HIV risk. A main feature of this study was that we examined HIV risk perception with naturalistic and ecologically valid stimulus materials. In a previous study [28], the stimulus materials depicted only the face of an unacquainted person while physical differences between the stimuli were minimized (neutral face expression, no background etc.). However, in face-to-face interactions, person perception includes a much wider array of information: Provocative or conservative clothing, tattoos, attire etc. are signatures of one's attitudes, behaviors, and group memberships, and these signatures can be expected to affect first impressions. Furthermore, the social environment in which people choose to portray themselves in photos may also exert influences upon impression formation. For instance, it has been shown that behavioral residues visible in the background, such as the tidiness, furniture style, and posters in one's office or flat, systematically influence person perception [30].

Second, we were interested in the neural correlates (ERPs) of perceived HIV risk for these naturalistic stimuli. Our hypothesis was that brain responses to persons varying in clothing, attire, and social environment are potent cues that trigger intuitive person perception. Intuitive processes are presumed to occur within split seconds, which sets them apart from the slower operations required for deliberation and elaborate analysis. Accordingly, brain responses were expected to be sensitive to perceived HIV risk at processing stages that are too early to be the product of elaborate stimulus analysis (i.e. <300 ms) [31], [32]. A further characteristic of intuition is its reliance on immediate affective reactions [16], [23]. Previous research has consistently revealed that affective stimulus processing is associated with enlarged late positive potentials between 300 and 700 ms after stimulus onset [33]. Thus, based on the notion that HIV risk is a potential threat for health, larger LPP amplitudes were expected for high HIV risk persons.

## Methods

### Participants

Sample 1: Forty volunteers (aged 20–32 years, M = 23.4, SD = 3.0, 12 males) were recruited on the campus of the University of Konstanz. In the first session, ERP recordings and explicit HIV risk perceptions were assessed for the stimulus set. In the second session, explicit HIV risk perceptions and personality trait ratings for the stimulus set were assessed. Participants received either 15 € or course credits as compensation. Three participants had to be excluded from analysis because of excessive EEG artifacts or an insufficient number of trials to compute ERP averages.

Sample 2: In order to obtain a greater data base and to examine the reliability of explicit person trait impressions, a second sample of 42 students of the University of Konstanz, aged 20–28 years (*M* = 23.7, *SD* = 2.4, 19 males) was recruited and provided explicit HIV risk perceptions and personality traits ratings for the stimulus set.

Participants provided written consent to the study protocol, which was approved by the Ethic Review Board of the University of Konstanz.

### Stimulus Materials

The stimulus set used in this study comprised photographs of persons in daily scenes. To assure high ecological validity, stimuli were selected based on the following six criteria: (1) A colored photo of a (2) single person located in the foreground, with (3) their face clearly visible. To be representative of the study's target population in terms of age and race, only photographs of (4) young (18–35 years old) (5) Caucasians were included. In order to resemble naturalistic viewing conditions and to facilitate impression formation, only (6) self-portraits exhibiting attire, socioeconomic status cues, or situational context features were included. Two stimulus sets were obtained, consisting of 120 female and 120 male persons. The photographs were retrieved with permission (creative commons) from a popular online photo-sharing community (www.flickr.com).

### Task and Procedure

The main study, including Sample 1, consisted of two sessions. Session 1 served to examine neural correlates of HIV risk perception. Towards this end, dense sensor ERPs were recorded while participants viewed 120 pictures of persons, each presented for 2 s and preceded by a fixation cross for 1 s. To increase ecological validity, each participant viewed 120 opposite sex persons. After a delay period of 1 s, participants were asked to evaluate how likely it is that the presented person is infected with HIV on a 7-point rating scale ranging from ‘very unlikely’ [1] to ‘very likely’ [7] (cf. [34]). The next trial was initiated after an ITI of 6.5 s.

In session 2, which took place within one week after the first session, participants from Sample 1 were presented with the same 120 target stimuli and asked to evaluate them according to the following seven trait characteristics: (1) attractiveness, (2) healthiness, (3) responsibility, (4) trustworthiness, (5) valence, (6) arousal, and (7) HIV risk. In addition, as proximal variable for behavior, participants rated (8) their willingness to interact with the person. All ratings were given on a 7-point scale, with greater numbers indicating that the respective characteristic is more pronounced.

Sample 2 rated the 120 stimulus persons on the same person characteristics (attractiveness, healthiness, responsibility, trustworthiness, valence, arousal, HIV risk, and willingness to interact with the stimulus person) as in Sample 1. All ratings were given on a 7-point scale.

### ERP Recordings and Analysis

Electrophysiological data were collected from the scalp using a 257-lead HydroCel Geodesic Sensor Net (EGI: Electrical Geodesics, Inc., Eugene, OR). The EEG was recorded continuously with a sampling rate of 250 Hz, with the vertex sensor as reference electrode, and on-line filtered from 0.1–100 Hz using Netstation acquisition software and EGI amplifiers. Impedances were kept below 50 kΩ, as recommended for this type of amplifier by EGI guidelines. Processing steps included low-pass filtering at 40 Hz, artifact detection, ocular artifact correction, bad sensor interpolation, baseline-correction for pre-stimulus (100 ms) ERP activity, and conversion to an average reference.

#### Risk categorization

**Figure 1:**
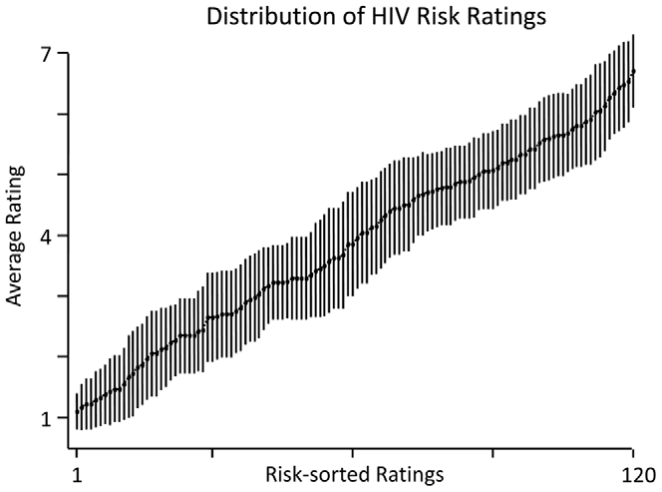
Average ratings of HIV risk and associated standard errors after rank-ordering each participant's ratings by HIV risk. Participants' ratings of HIV risk varied across the full range of the scale (1 - low HIV risk; 7 - high HIV risk).

In order to calculate ERPs toward high and low HIV risk stimulus persons, it is necessary that the presented persons varied in their ascribed HIV risk. To determine the distribution of the given risk ratings, risk ratings provided by Sample 1 in Session 1 were rank ordered for each participant and the mean HIV risk rating for each rank was calculated across participants. As shown in Figure 1, mean HIV risk ratings ranged from very low HIV risk (minimum = 1.1) to very high HIV risk (maximum = 6.7). Additional information is provided by calculations of the variance and range of the HIV risk ratings for each participant. On average, HIV risk ratings showed substantial intra-individual variance (mean variance = 2.5) and the full range of the risk scale was used by the participants (mean range = 5.6). As expected, variation in ascribed HIV risk was similar in Session 2 (minimum = 1.18, maximum = 6.6, mean variance = 2.2, mean range = 5.4). Inter-rater agreement for the rated HIV risk was high, with intra-class correlations (two-way random, mean) of ICC = .93 for female raters and ICC = .95 for male raters. The test-retest reliability across Session 1 and Session 2 was high with *r* = .87 (*p*<.001). These analyses demonstrate that our naturalistic stimuli produced broad variations in perceived HIV risk and that risk ratings were highly stable across a time lag of one week. To calculate ERP averages, stimulus persons were categorized according to the idiosyncratic risk ratings assessed in Session 1. Specifically, stimulus persons receiving HIV risk ratings between 1 and 3 were coded as ‘low’ HIV risk (mean across subjects = 2.27, SD = 0.26) and stimulus persons receiving HIV risk ratings between 5 and 7 were coded as ‘high’ HIV risk (mean = 5.49, SD = 0.22). Importantly, the ERP findings reported in the following remained virtually unchanged when z-standardized risk ratings for each participant were used instead of the raw HIV risk rating scores, (low HIV risk: z<−0.2; high HIV risk: z>0.2).

#### Area score assessment

Two ERP components sensitive to HIV risk were identified by visual inspection and single sensor waveform analysis (cf. [27]). In a time interval between 220–340 ms post stimulus, the fronto-central component (low vs. high HIV risk) was scored including EGI sensors #218, 219, 220, 225, 226, 227, 228, 230, 231, 232, 233, 234, 235, 236, 237, 238, 239, and 240 (right) and #61, 67, 73, 241, 242, 243, 244, 245, 246, 247, 248, 249, 250, 251, 252, 253, 254, and 255 (left; see Fig. S1). The effect appeared reversed in polarity over occipito-temporal sites and was assessed by collapsing across the following sensors #127, 128, 138, 139, 140, 141, 148, 149, 150, 151, 152, 158, 159, 160, 161, 168, 169, and 129 (right), and #97, 98, 99, 100, 106, 107, 108, 109, 110, 114, 115, 116, 117, 118, 123, 124, 125, and 136 (left). The centro-frontal LPP component was indexed as mean activity from 350–700 ms comprising right (#5, 6, 7, 184, 185, 196, 197, 198, 206, 207, 214, 215, and 224) and left (#24, 30, 42, 51, 52, 44, 43, 17, 16, 23, 29, 36, and 41) EGI sensors (see Fig. S1). The early ERP components were submitted to a repeated-measures ANOVA including the independent variables ‘HIV Risk’ (low vs. high), ‘Location’ (fronto-central vs. occipito-temporal), and ‘Laterality’ (left vs. right). The late ERP component was entered in ANOVA analysis including the independent variables of ‘HIV Risk’ and ‘Laterality’. Where appropriate, degrees of freedom were adjusted using the Greenhouse–Geisser method to correct for violations of sphericity.

## Results

### First Impressions and HIV Risk: Perceived Personality Traits

Factor analyses of the assessed personality traits were conducted to examine personal impressions relating to intuitive HIV risk perception. Specifically, the eight explicit ratings for each stimulus person assessed in Sample 1, during the second session, and in Sample 2, i.e., attractiveness, healthiness, responsibility, trustworthiness, valence, arousal, HIV risk, and willingness to interact with the stimulus person, were analyzed using principal component analyses (PCA with varimax rotation). For the unit of analysis, averaged responses towards each stimulus person across both studies were used. The number of factors to be extracted was determined by three criteria: Cattell's scree test, the parallel analysis of the eigenvalues (PA), and Velicer's minimum average partial test (MAP) (cf. [35]). The scree test showed a substantial drop in the eigenvalues after two factors. The parallel analysis of the eigenvalues also suggested the extraction of two factors. Specifically, the first two eigenvalues from the respective actual data set (4.40, 2.58, 0.36) were greater than the eigenvalues derived from the respective random data set (1.28, 1.17, 1.09). Furthermore, the MAP test also indicated the retention of two factors.

The first factor, which accounted for 55% of the variance, had a strong positive relationship with attractiveness, valence, healthiness, and willingness to interact, and can therefore be interpreted as ‘valence-approach’ factor. The second factor, with 32% of explained variance, had a high negative loading for perceived HIV risk and perceived arousal, while trustworthiness and responsibility had a high positive loading. Thus, factor 2 appears to capture the perceived ‘safeness’. Importantly, the two factor structure revealed a clear dissociation among measures of the ‘valence-approach’ and ‘safeness’ dimension. To obtain a solution unbiased with respect to risk, all trait ratings except perceived risk were submitted to a second PCA factor analysis (cf. [36]). Perceived risk was highly and positively correlated with the second factor (*r* = .85, *p*<.001) and correlated negatively and only to a small degree with the first factor (*r* = −.13, *p*<.01).

**Figure 2:**
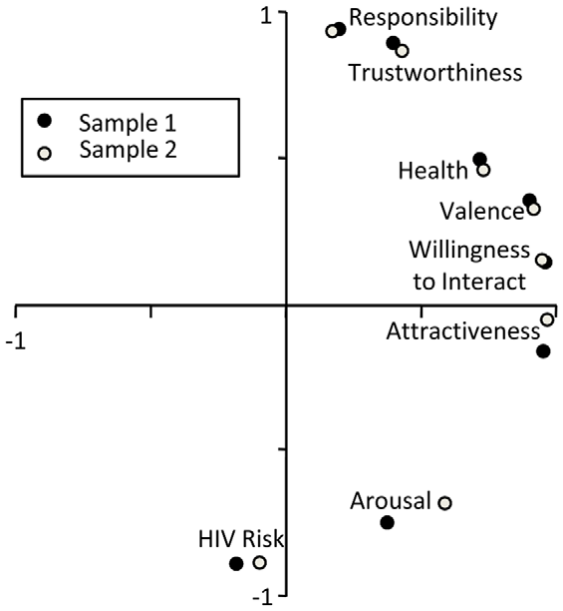
Factor loadings of explicit person impression ratings (PCA, x-axis represents factor 1, y-axis factor 2) from Sample 1 and Sample 2.

To further test the robustness of the two-dimensional solution, additional factor analyses were conducted separately for Sample 1 and Sample 2. The solutions within the two separate samples were remarkably similar (see Fig. 2). Again, all three criteria for the number of factors to extract (Cattell's scree test, PA, and MAP) indicated two factors to extract in both samples. The first factor explained 56% of the variance in both Sample 1 and Sample 2 and the second factor explained 33% and 31%, in Sample 1 and Sample 2, respectively. In both samples, judgments of attractiveness, valence, healthiness, and willingness to interact had high positive loadings on the first factor, while judgments of HIV risk, trustworthiness and responsibility had high loadings on the second factor. Thus, the basic factor structure remained virtually the same across both samples, confirming the robustness of the observed solution.

### Intuitive Risk Perception: ERPs

#### Fronto-central and occipito-temporal component (220–340 ms)

**Figure 3:**
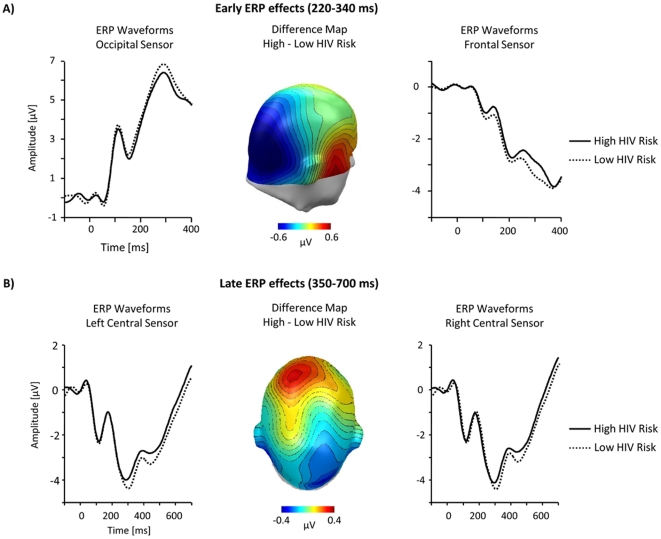
Relationship between HIV Risk ratings and ERPs. (A) Representative ERP-waveforms for high and low risk stimuli over occipital (left panel) and frontal (right panel) sensor sites. The scalp potential map shows the topography of the difference between the high and low risk stimuli averaged across the time window from 220–340 ms (middle panel). (B) Representative left and right centro-frontal sensor sites illustrate the late ERP effect. The difference scalp map (high - low risk) shows the topography of the risk modulation in the LPP time window (350–700 ms).

As illustrated in Figure 3A, the present study obtained evidence for a relatively early modulation of the ERP waveform by HIV risk. Overall, the ERP waveform presents a positive polarity over posterior sensors and a negative polarity over anterior sites. However, the encoding of risky stimulus persons resulted in a relative negative shift in the ERP waveform over occipito-temporal sensor regions and a corresponding shift over fronto-central sensor sites. The topography of the differential ERP activity (i.e., a relative posterior negativity and anterior positivity) for high HIV risk is further illustrated by the calculation of difference maps (high – low HIV risk; see Fig. 3A middle panel). Substantiating these observations, the overall ANOVA analysis revealed a significant interaction of ‘HIV Risk×Location’, *F*(1,36) = 6.6; *p*<0.05, partial η^2^ = 0.15, ε = 1, indicating that the effects of the variable ‘HIV Risk’ appeared with reversed polarity over fronto-central and occipito-temporal sites. Accordingly, separate ANOVAs were calculated for fronto-central and occipito-temporal regions. Over fronto-central leads, a main effect of ‘HIV Risk’ was observed, *F*(1,36) = 5.0, *p*<0.05, partial η^2^ = 0.12, ε = 1, indicating a less negative potential for high HIV risk (*M* = −2.9, *SD* = 1.7) compared to low HIV risk persons (*M* = −3.3, *SD* = 1.9). Over occipito-temporal sites, the HIV risk effect reversed in polarity, *F*(1,36) = 6.3, *p*<0.05, partial η^2^ = 0.15, ε = 1. High HIV risk persons (*M* = 5.2, *SD* = 2.4) elicited a less positive potential compared to low HIV risk persons (*M* = 5.5, *SD* = 2.6). No effects involving the variable ‘Laterality’ reached significance in these analyses.

#### Centro-frontal component (350–700 ms)

A second modulation of the ERP by perceived HIV risk status appeared in a time window between 350 and 700 ms over centro-frontal sensor sites. Considering the differential ERP activity (high - low HIV risk), which is illustrated in Figure 3B (middle panel), shows that the processing of risky stimuli is associated with a relative positive potential over centro-frontal sensor sites. Statistical analysis confirmed significant differences for high (*M* = −1.1, *SD* = 1.7) and low HIV risk stimulus persons (*M* = −1.3, *SD* = 1.7); ‘HIV Risk’ *F*(1,36) = 5.3, *p*<0.05, partial η^2^ = 0.13, ε = 1. While the effect appeared to be more pronounced over midline and left sensor sites (see Fig. 3B), the interaction of ‘HIV Risk×Laterality’ was not significant, *F*(1,36) = 0.4, ns.

#### Control analyses

Factor analysis suggests that perceived HIV risk is reliably associated with trait characteristics such as trustworthiness and responsibility while being distinct from judgments of attractiveness, valence, and willingness to interact. Based on these findings, a set of control analyses was conducted to determine the specificity of the HIV risk - ERP findings. Specifically, the judgments of attractiveness, healthiness, valence, arousal, willingness to interact, responsibility, and trustworthiness obtained in the second session within Sample 1 were sorted into low and high categories and analyzed for ERP differences. With regard to the early ERP component (220–340 ms), trustworthiness, responsibility, and arousal revealed significant effects in the frontal Fs(1,36)>6.5, p<0.05, partial η^2^>0.15, ε = 1) and occipital sensor clusters, Fs(1,36)>4.7, p<0.05, partial η^2^>0.12, ε = 1), mirroring the effects observed for HIV risk. Specifically, individuals evaluated as low in trustworthiness and responsibility and high in arousal elicited an increased occipital negativity and frontal positivity. In contrast, ratings of attractiveness, valence, health, and willingness to interact showed no significant modulation of the early ERP component. The association of the later ERP component (350–700 ms) with these additional judgments was generally less pronounced and failed to reach significance. Further analysis revealed that trustworthiness showed a significant effect in this time window with a more lateralized left and right fronto-central sensor cluster (F(1,36) = 4.2, p<0.05, partial η^2^ = 0.10, ε = 1). The ERP findings, in particular with regard to the early component, further corroborate the hypothesis that judgments of HIV risk, trustworthiness, and arousal share a substantial part of their variance, presumably reflecting common meaning structures.

## Discussion

Rather than relying on effective strategies for risk prevention (i.e., consistent condom use), people may rely on their intuition to identify potential sexual partners high at risk for sexually transmitted diseases. Investigating the operation and nature of this intuitive mode of risk perception is important as this strategy does not provide adequate protection [8], [10]. The present study revealed two noteworthy findings: First, the ERP findings demonstrate features of stimulus significance and speed in intuitive HIV risk perception using ecologically valid stimulus materials, i.e., portraits varying in clothing, attire, and situational context. Second, intuitive HIV risk perception seems to be related to key dimensions of the high at risk stereotype, i.e., perceived responsibility and trustworthiness. These findings suggest that a brief glimpse of an unacquainted person can be sufficient to form an impression of others' HIV risk status, which presumably reflects the activation of a broader associative person network related to safeness vs. dangerousness of interpersonal relationships.

### The Risk Heuristic

It has been proposed that risk perception can be based on intuitive rather than rational stimulus analysis. Specifically, intuitive risk perceptions may be based on negative or positive reactions towards stimuli, which are experienced as a feeling state [16]. In accordance with this notion, a ‘valence-approach’ factor was found in the both samples. Specifically, valence, attractiveness and perceived healthiness were strongly related to the behavioral approach dimension ‘willingness to interact’, constituting a ‘valence-approach’ factor. However, rather than being related to the ‘valence-approach’ dimension, the results strongly suggest that HIV risk ratings relate to impressions of responsibility and trustworthiness, comprising a second factor. Thus, risk perceptions do not appear to be comprehensively captured by valence or physical attractiveness. In a previous study using standardized faces as stimulus material, perceived HIV risk and attractiveness were also shown to represent distinct aspects of person perception, with a dissociation in the brain signature of explicit HIV risk and attractiveness ratings [28].

Furthermore, perceived risk does not simply mirror perceived health, since health was more strongly associated with the ‘valence-approach’ factor than with the ‘safeness’ factor. Accordingly, perceived health appears to be linked with attractiveness and approach tendencies as assumed by the ‘good genes sexual selection theory’ [37] but is obviously not a proximal variable for perceived HIV risk. One may speculate that the stronger link of perceived HIV risk with responsibility/trustworthiness rather than health/attractiveness is specific to sexually transmitted diseases (STDs). The risk for STDs is behavioral in origin and, thus perceived as being largely under individual control. In this case, the individual is seen as being mainly responsible for handling his or her own risks to health, and as a result, perceived responsibility and trustworthiness may become key cues for inferring riskiness.

A main finding of the present study is that HIV risk, trust, and responsibility loaded on a common factor related to safeness in interpersonal relationships. These findings indicate the activation of a high at risk stereotype. Specifically, a low sense of responsibility and distrust was reliably named as a key feature characterizing persons with a high risk of HIV [cf. 22]. Importantly, these person based characteristics seem highly amenable to first impressions in person perceptions. For instance, previous research has firmly established that trustworthiness could be inferred from facial appearance spontaneously and with minimal processing time [38], [39]. Thus, one may speculate that the intuitive perception of HIV risk is based on first impressions about trustworthiness and responsibility (cf. [14]). A noteworthy feature is that both using appearance-based cues to infer trait characteristics, and relying on this information to gauge HIV risk, seems to operate at the implicit level. Specifically, when probed at the end of the experiment, most participants could not state which information they had based their HIV risk estimates on. Overall, with regard to the perception of others' HIV risk status, intuition seems to be based on implicit stereotype representation distinct from the good-bad dimension. This is also reflected in the ERP findings showing that these substantially correlated ratings, HIV risk, trustworthiness, responsibility, and arousal, lead to a similar assignment of EEG epochs into e.g., risky/safe or untrustworthy/trustworthy categories. Specifically, analyzing the ERP data based on dichotomized ratings of trustworthiness, responsibility, and arousal elicited ERP modulations that were similar to HIV risk ratings with regard to the early ERP component (220–340 ms).

### Brain Correlates of Intuitive Risk Perception

The present study revealed that naturalistic photos of unacquainted persons elicited brain correlates of intuitive HIV risk perception. Taking naturalistic portrayals of persons as stimulus material ensured that the photos conveyed individual behavioral residuals and attitudes through multiple channels, such as clothing, attire, and social context, which are all crucial for impression formation in everyday life. However, the stimulus material was based not only on idiosyncratic self-presentation (‘This is who I am’) but also the perception of high or low HIV risk as based on the individual ratings provided by the participants, rather than a priori or normative category assignment.

This experimental procedure builds upon the conception that the perception of unacquainted individuals is based on an interconnected associative network structure containing stimulus, response, and meaning elements [40]. Implicit learning provides a means of associating stimulus and meaning elements with regard to key characteristics of person perception [41]. Furthermore, intrinsic stimulus significance may be represented in the network structure that possesses more and stronger connections, proposed to lead to differential brain responding to high and low HIV risk. Consistent with this notion, the findings revealed a modulation of the LPP component, which has been established in numerous previous studies as a reliable brain marker of affect and intrinsic stimulus significance [33]. Specifically, the difference between people evaluated as high and low in HIV risk was reflected in larger LPP amplitudes over centro-parietal sensor sites in a time window from 350–700 ms for the high risk stimulus category. Thus, the LPP findings demonstrate the feature of intrinsic stimulus significance, which is characteristic for an intuitive processing mode.

A further characteristic of intuition is its remarkable speed. Intuitive processing is assumed to reflect a fast processing mode, which utilizes unconsciously generated inferences [23]. Determining the onset of differential brain responses to high and low risk stimulus category provides an upper boundary when risk related information is extracted. The relatively early onset of differentiation among high and low risk categories supports the notion of processing efficiency (∼220 ms) and is too short for deliberate reasoning to play a role. Interestingly, the onset of the differential brain responses to the high risk stimulus category was somewhat delayed (∼40 ms) in the present study in comparison to a previous study which presented standardized images of faces. However, despite a later onset, early differential brain responses to the high risk stimulus category appeared considerably longer for naturalistic photographs of persons in comparison to standardized faces (120 ms vs. 60 ms). Thus, while the extraction of risk related information from photographs of persons varying in clothing, attire, and situational context seems to demand longer processing time, the effect was more sustained over time. Overall, the present findings showed that naturalistic pictures of persons elicit the brain signature of two core features of intuition, i.e. intrinsic stimulus significance and speed of processing.

### Risk Perception: A Broader Perspective

The finding that people report that they often ‘just know’ whether a person is risky or safe provides the starting point for the scientific analysis of the intuitive sensing of risk in an important domain of health psychology. Evidence is accumulating that risk impressions can be formed with little processing time and that people are unable to provide an explanation for the perceived feeling [16], [24]. Neural measures seem well suited to make such key features of intuition amenable to scientific investigation by tracking the time course of person perception. However, it has to be pointed out that participants in the present study were not selected based on whether they relied on this illusory control strategy and that these findings do not imply that our participants actually make judgments about others' HIV risk status within a fraction of a second. What the present findings do demonstrate is that the task of explicitly forming an impression about HIV risk is sufficient to reveal an intuitive mode of risk perception, which seems to operate via rapid and largely automatic processing routines. Supporting this notion, a recent study revealed systematic ERP differences related to low and high HIV risk categories in an implicit experimental condition [42].

Neural measures of intuition were complemented in this study with self-report data concerning important person characteristics revealed by previous research. HIV infection is not reliably associated with overt signs, and at first glance, it appears puzzling that participants' explicit ratings are systematically related to preceding ERP components. The present findings may provide a solution to the puzzle by indicating that participants' HIV risk ratings systematically relate to trustworthiness and responsibility, which are key characteristics of the high risk HIV stereotype, and importantly, can be extracted from faces rapidly and with ease. The understanding of the operation and nature of intuitive processes in risk perception provides an important base for future research aiming at strategies promoting the adoption of effective precautionary behaviors. For instance, informing participants about how easily erroneous beliefs about their partners' safety are formed and providing direct experiential experience via corrective feedback information may provide new avenues for intervention (cf. [8]). Overall, it is proposed that the integration of methods and findings across various domains in psychology is helpful for furthering our understanding of intuitive processes pertaining to risk perception [24].

### Summary

In the present study, we took a novel approach to shed light on the processes involved in HIV risk perception. Using a combination of self-report and neuroscientific measures, our results reveal how intuitive brain mechanisms can lead to snap impressions about HIV risk, and how these impressions are embedded in a set of related, trait characteristics pertaining to a high HIV risk stereotype. These findings are important because intuitive processes may lead people to believe that they know who poses a risk and to underestimate their own risk of becoming infected with HIV. Taken together, these findings provide empirical evidence for theoretical models of risk perception, such as the ‘risk as feelings’ notion [24].

## Supporting Information

Figure S1 **Illustration of the sensor-montage of the high-density EEG-system.** Grey areas indicate sensor clusters included in conventional ANOVA analysis of the frontal, occipital, and fronto-central components.
(TIF)
