# Supplementary figures and images for: First Impressions of HIV Risk: It Takes Only Milliseconds to Scan a Stranger
Source: PLoS One. 2012 Jan 24;7(1):e30460. doi: 10.1371/journal.pone.0030460 (PMC3265480; doi:10.1371/journal.pone.0030460)

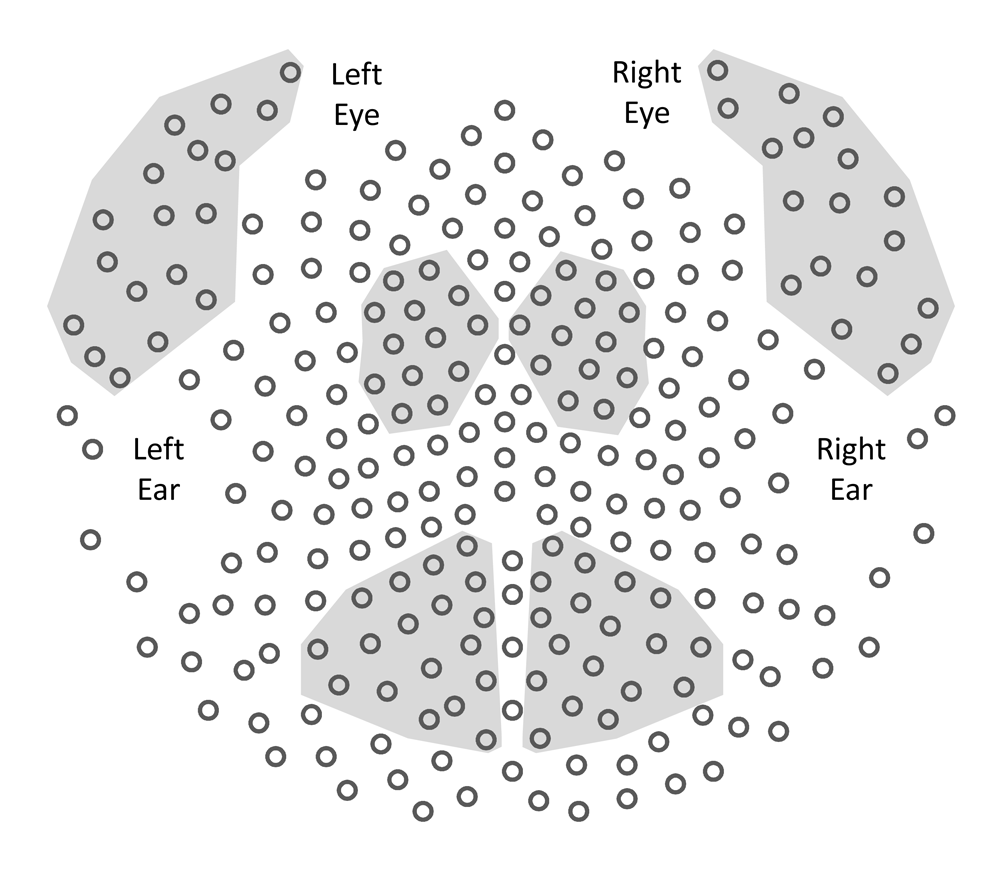

Supplement: Figure S1 — Illustration of the sensor-montage of the high-density EEG-system. Grey areas indicate sensor clusters included in conventional ANOVA analysis of the frontal, occipital, and fronto-central components. (TIF) [file pone.0030460.s001.tif]
